# Human mobility under disasters: a systematic review and framework for equitable and resilient mobility governance

**DOI:** 10.1038/s44304-025-00153-9

**Published:** 2025-11-07

**Authors:** Fengjue Huang, Junqing Tang, Pengjun Zhao, Zhihe Chen, Jiaying Li, Wei Lyu

**Affiliations:** 1 School of Urban Planning and Design, Shenzhen Graduate School, Peking University, Shenzhen, China; 2 Key Laboratory of Earth Surface System and Human-Earth Relations of the Ministry of Natural Resources of China, Shenzhen Graduate School, Peking University, Shenzhen, China; 3 Center for Transport Studies, Department of Civil and Environmental Engineering, Imperial College London, London, UK; 4 Science and Solutions for a Changing Planet Doctoral Training Partnership, Imperial College London, London, UK; 5 Department of Civil and Environmental Engineering, Tufts University, Medford, MA USA

**Keywords:** Complex networks, Complex networks, Environmental studies, Geography, Geography

## Abstract

Global crises, including climate-induced disasters and health emergencies, are disrupting human mobility, making it critical to understand population movements for effective planning. Here, we systematically review 946 studies, framing mobility as simultaneously responding to external shocks and transmitting impacts. Our analysis first maps the field’s methodological and geographic landscape before focusing on three dimensions: (1) universal response patterns to external shocks, (2) structural inequalities mediating these responses, and (3) cascading effects from mobility to other interconnected systems. We identify predictable temporal and spatial dynamics in human mobility responses driven by adaptive behaviors and psychological factors. Ultimately, these responses are filtered through vulnerability pathways determined by income, race, gender, and disability status, transmitting cascading effects across environmental, health, and economic systems. Based on the review findings, we propose the FAIR-HEART framework for equitable mobility governance and discuss the future directions, providing actionable guidance for building resilient societies.

## Introduction

The resilience and functionality of urban landscapes are fundamentally contingent on the dynamics of human mobility^1^. This mobility, defined as the spatial and temporal movement of populations, is undergoing profound transformations in response to an unprecedented convergence of global crises. Climate-related disasters, infectious disease outbreaks, and geopolitical conflicts have collectively reshaped patterns of human mobility, with far-reaching implications for safety, survival, and societal stability^2–6^. Between 2014 and 2023, disasters displaced an average of 24 million people annually, with 92% triggered by climate-related hazards, including floods, storms, droughts, and wildfires^7^. The consequences of displacement above are severe, with climate-related events alone causing over $2.24 trillion in economic losses and 1.3 million deaths between 1998 and 2017^8^. Importantly, mobility responses extend far beyond simple displacement. The COVID-19 pandemic sharply reduced global mobility, with a 74% decline in travel^9^. The Russia-Ukraine conflict led to an 84% reduction in nighttime light intensity (i.e., an important indicator of human mobility) across affected regions^10^. Taken together, these cases highlight the systemic vulnerability of mobility systems: once destabilized by crises, they can deteriorate rapidly, leading to severe economic and human losses.

As disasters become more frequent and severe, understanding human mobility patterns during emergencies has become essential for effective disaster preparedness and adaptive governance. A central premise in this understanding is that human mobility constitutes a predictable system-level response to external shocks, while simultaneously serving as a transmitter of impacts to interconnected systems. Specifically, human mobility functions as a responsive system when populations adapt their movement behaviors in reaction to external threats, encompassing immediate evacuation and temporary displacement as well as longer-term migration and shelter-in-place immobility caused by flash floods^11,12^. At the same time, mobility operates as a transmitter, often triggering cascading effects that amplify risks and vulnerabilities across interconnected social, economic, and environmental systems^13^. Recognizing this dual role is crucial for holistic disaster planning and urban planning.

Despite the growing recognition of mobility’s criticality in disaster responses, many previous studies remain trapped within disciplinary silos and event-specific case studies^14–16^. Moreover, while individual studies have documented mobility inequities and cascading system effects^17–19^, these crucial insights remain critically fragmented. Such fragmentation hampers our capability to anticipate and mitigate the compound vulnerabilities that emerge when disasters intersect with deep-seated social and economic disparities. Although diverse fields have generated valuable insights from their respective vantage points, their perspectives rarely converge into a unified understanding of mobility as a dynamic system that both responds to and transmits the impacts of crises. The absence of a holistic, cross-system perspective has impeded the identification of universal patterns that transcend disaster types and geographic contexts, thereby limiting the development of generalizable, robust policy frameworks.

Several recent reviews have synthesized mobility research from different perspectives. Wang et al. mapped the connections between mobility research and a wide array of urban challenges^20^. Pappalardo et al. provided a historical perspective on the discipline’s evolution^21^, while the IMAGE framework by Zhang et al. offers a comprehensive bibliometric analysis of research trends^22^. While these foundational syntheses are essential for understanding mobility under normal conditions, they do not specifically focus on the disaster-induced human dynamics and multi-disaster scenarios. Consequently, we still lack a cohesive, cross-disaster framework that integrates the full range of mobility responses from evacuation to shelter-in-place immobility, especially from the perspective of responsive and transmissive aspects.

To address the pressing needs above, this systematic review makes three core contributions. First, we introduce a new conceptual lens for understanding human mobility in emergencies: framing mobility as a dual-role system that is simultaneously a responsive system adapting to external shocks and a transmissive system actively driving cascading impacts across interconnected environmental, health, and economic domains. Second, by synthesizing 946 studies published between 2019 and 2025, our work overcomes the fragmentation of previous event-specific case studies to provide a new systematic synthesis, identifying universal response patterns that cut across diverse disaster types, geographical regions, and socio-cultural contexts. Third, building upon this synthesis, we propose the FAIR-HEART framework, an operational and cyclical governance model specifically designed to manage mobility’s dual role and provide an actionable tool for policymakers.

**Fig. 1:**
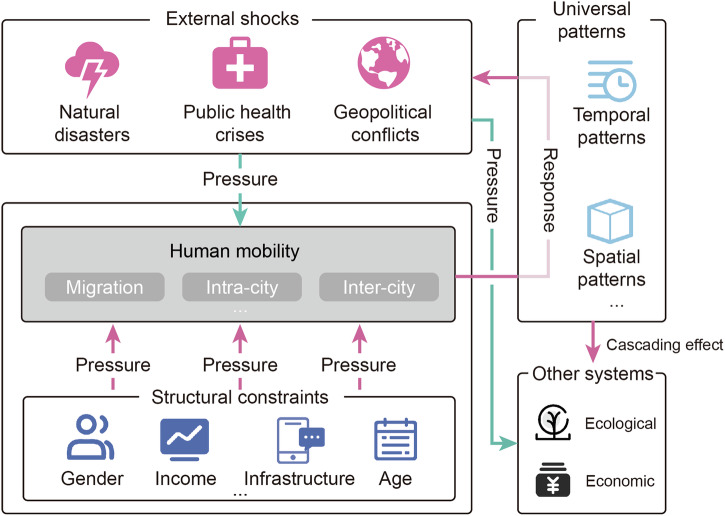
Conceptual basis for the analytical approach. Figure created using Adobe Illustrator, version 28.7.9.

To substantiate these contributions, our synthesis is structured along the following three dimensions (Fig. 1), which directly build upon the dual function of mobility as both a responsive and transmissive system:

1. Universal response patterns: Routine human mobility exhibits remarkable and predictable regularities^23–25^. A foundational question for disaster governance is whether similar predictable patterns emerge under the duress of extreme events. To answer this question, we first identify universal patterns of mobility response, revealing predictable ways populations react to external shocks.
2. Manifested inequities: Disasters do not affect all populations equally^26^. Therefore, our analysis next examines how pre-existing structural inequities, rooted in socioeconomic status, gender, disability, and age, channel disaster impacts by creating differentiated mobility capacities and opportunities.
3. Cascading effects across systems: Recognizing that human movement is not a passive outcome but an active driver of systemic change, our synthesis finally examines mobility’s transmissive function by analyzing how altered movement patterns transmit cascading impacts across interconnected environmental, health, and economic systems.

The proposed FAIR-HEART framework translates these synthesized insights into an actionable model for equitable and resilient mobility governance. It integrates nine principles: Flexibility, Accessibility, Inclusivity, Resources, Harmonization, Education, Anticipation, Reversal, and Technology. Rather than imposing a one-size-fits-all solution, FAIR-HEART provides a flexible foundation that can be tailored to local contexts and vulnerability profiles. With this framework, we provide an essential, evidence-based tool for policymakers and practitioners to design mobility governance strategies that address both the responsive and transmissive dimensions of disaster-induced movement, moving beyond infrastructure-focused resilience to a more holistic, human-centered approach to planning under uncertainty.

## Results

### Overall landscape of existing studies

**Fig. 2:**
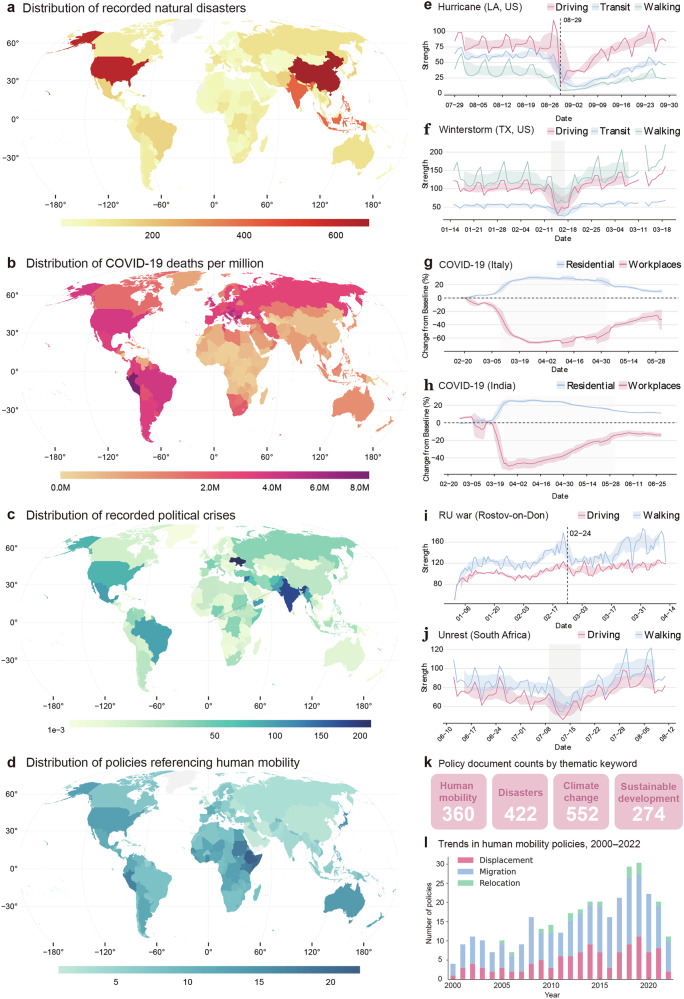
Global disaster distribution, mobility responses, and policy landscape. Spatial distribution of recorded natural disasters (**a**), COVID-19 deaths per million (**b**), and political crises (**c**). **d** Distribution of policies referencing human mobility. **e**–**j** Temporal mobility patterns (driving, transit, walking) during specific disaster events: Hurricane Laura, Louisiana, US (**e**); Winter Storm Uri, Texas, US (**f**); COVID-19 lockdowns in Italy (**g**) and India (**h**); Russia-Ukraine war, Rostov-on-Don (**i**); and civil unrest, South Africa (**j**). **k** Policy document counts by thematic keywords. **l** Temporal trends in human mobility policies by category, 2000–2022. (Note: regenerated using data from Apple mobility and the United Nations). Figure created using RStudio, version 2024.12.1 + 563.pro5, and Adobe Illustrator, version 28.7.9.

Our analysis begins by mapping the overall landscape of research on human mobility under disasters. Figure 2 illustrates the global distribution of recent crises and policy responses, which provides the context for this review. To dissect the field’s architecture, we analyze the geographic and methodological distribution of the reviewed studies across six key facets (see Supplementary Table S1 for detailed definitions): data type, disaster type, spatial granularity, mobility metrics, modeling approaches, and geographic regions (Table 1). This analysis reveals a field characterized not by global uniformity, but by significant regional disparities in research capacity, thematic focus, and analytical priorities, which we detail below.

**Table 1:** Mobility data characteristics by regions

Characteristic	Africa, *N* = 126	Americas, *N* = 303	Asia, *N* = 317	Europe, *N* = 237	Oceania, *N* = 75
Type of data					
Social media data	12 (9.5%)	41 (13.5%)	30 (9.5%)	27 (11.4%)	9 (12.0%)
Mobile phone data	30 (23.8%)	129 (42.6%)	116 (36.6%)	83 (35.0%)	21 (28.0%)
Census/migration	49 (38.9%)	127 (41.9%)	78 (24.6%)	79 (33.3%)	25 (33.3%)
Flight/traffic data	8 (6.3%)	71 (23.4%)	58 (18.3%)	42 (17.7%)	5 (6.7%)
Simulation	4 (3.2%)	10 (3.3%)	23 (7.3%)	10 (4.2%)	1 (1.3%)
Other	52 (41.3%)	82 (27.1%)	107 (33.8%)	71 (30.0%)	26 (34.7%)
Type of disasters					
Natural hazards	59 (46.8%)	93 (30.7%)	92 (29.0%)	34 (14.3%)	26 (34.7%)
Technological/Man-made hazards	1 (0.8%)	5 (1.7%)	18 (5.7%)	2 (0.8%)	0 (0.0%)
Health emergencies	57 (45.2%)	200 (66.0%)	191 (60.3%)	174 (73.4%)	44 (58.7%)
Socio-political crises	12 (9.5%)	11 (3.6%)	20 (6.3%)	31 (13.1%)	5 (6.7%)
Granularity					
Individual-level movements	21 (16.7%)	81 (26.7%)	99 (31.2%)	65 (27.4%)	11 (14.7%)
Aggregate with population details	37 (29.4%)	80 (26.4%)	82 (25.9%)	62 (26.2%)	14 (18.7%)
Aggregate without population details	30 (23.8%)	109 (36.0%)	97 (30.6%)	65 (27.4%)	24 (32.0%)
mixed	22 (17.5%)	64 (21.1%)	48 (15.1%)	44 (18.6%)	10 (13.3%)
Unknown granularity	28 (22.2%)	30 (9.9%)	33 (10.4%)	26 (11.0%)	19 (25.3%)
Mobility metric					
Volume	65 (51.6%)	213 (70.3%)	186 (58.7%)	152 (64.1%)	41 (54.7%)
Time	25 (19.8%)	120 (39.6%)	115 (36.3%)	65 (27.4%)	14 (18.7%)
Spatial	40 (31.7%)	131 (43.2%)	124 (39.1%)	75 (31.6%)	20 (26.7%)
Network	43 (34.1%)	100 (33.0%)	117 (36.9%)	86 (36.3%)	24 (32.0%)
Contextual	40 (31.7%)	110 (36.3%)	93 (29.3%)	62 (26.2%)	21 (28.0%)
Model					
Statistical & Econometric	57 (45.2%)	172 (56.8%)	147 (46.4%)	135 (57.0%)	35 (46.7%)
Spatial & Network-analytic	25 (19.8%)	119 (39.3%)	98 (30.9%)	59 (24.9%)	13 (17.3%)
Simulation & Agent-based	10 (7.9%)	27 (8.9%)	39 (12.3%)	16 (6.8%)	4 (5.3%)
Machine-learning & Clustering	21 (16.7%)	64 (21.1%)	73 (23.0%)	42 (17.7%)	14 (18.7%)
Qualitative & Theoretical	58 (46.0%)	82 (27.1%)	86 (27.1%)	66 (27.8%)	28 (37.3%)

Percentages presented are by regions (that is, column-wise proportions). Detailed definitions of all categories are provided in Supplementary Table S1.

#### Data types and availability in different regions

The distribution of data sources across regions reveals persistent technological asymmetries. Mobile phone data dominates research in the Americas (42.6%) and Asia (36.6%), while African studies rely more heavily on traditional census/migration data (38.9%). This bifurcation reflects not merely methodological choice but fundamental disparities in research infrastructure. Europe occupies an intermediate position, balancing mobile data usage (35.0%) with alternative sources (30.0%), mirroring its diverse technological landscape. Despite global advances in mobile technology, research capabilities remain unevenly distributed, potentially skewing our understanding of global mobility patterns during emergencies.

#### Region-specific risk profiles

Regional vulnerability patterns emerge clearly through research focus distribution. African studies divide attention between natural hazards (46.8%) and health emergencies (45.2%), while European studies emphasize health emergencies (73.4%) with relatively little focus on natural hazards (14.3%). This disparity likely reflects different regional priorities, but also raises questions about potential research gaps. Asia’s comparatively higher focus on technological and man-made hazards (5.7%) reflects the region’s extensive nuclear infrastructure and high population densities, which heighten vulnerability to technological failures and necessitate effective mass evacuation planning. Europe’s greater attention to socio-political crises (13.1%) reflects ongoing migration pressures and complex geopolitical dynamics. These patterns suggest research agendas are shaped more by regional threat landscapes than by standardized global protocols.

#### Mobility metric selection reveals epistemic priorities

Regional preferences in mobility metrics expose underlying epistemic frameworks. Volume-based metrics dominate across all regions, especially in the Americas (70.3%), suggesting a bias towards quantification. Asia and European studies demonstrate pronounced preference for network metrics, reflecting emphasis on connectivity patterns rather than mere movement volumes. These regional variations in emergency mobility research reveal fundamental disparities in technological infrastructure, research priorities, and epistemological frameworks. Addressing these gaps requires not merely technological transfer but also a critical examination of how regional research agendas reflect and respond to differentiated vulnerability landscapes.

### Universal response patterns

**Fig. 3:**
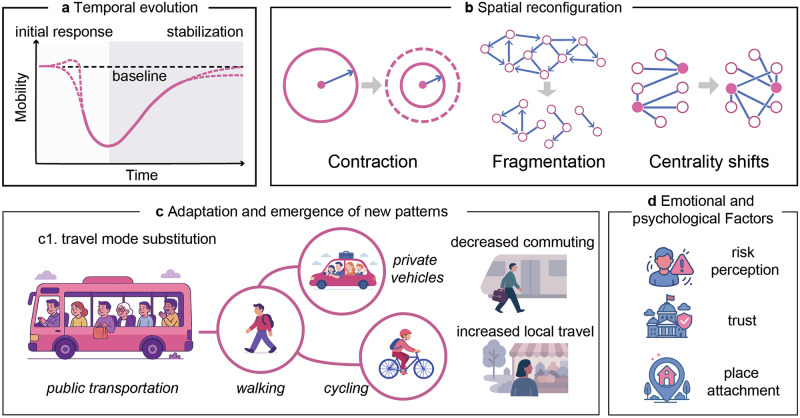
Schematic drawings of universal patterns in human mobility during emergencies. Crises disrupt human mobility across multiple dimensions. **a** Temporal evolution is marked by an abrupt deviation from baseline movement patterns during the initial response phase, followed by gradual stabilization. **b** Spatial reconfiguration includes contraction of travel range, fragmentation of mobility networks, and shifts in network centrality as movement concentrates around essential services or safe zones. **c** Adaptation and emergence of new patterns reflect behavioral innovations such as travel mode substitution (e.g., from public transit to walking, cycling, or private vehicles), altered commuting behavior, and localization of travel. **d** Emotional and psychological factors—including risk perception, trust, and place attachment—critically shape whether, how, and when people move. Together, these dimensions underscore the complex, multi-layered dynamics of human mobility in response to disruptive events. Figure created using Adobe Illustrator, version 28.7.9.

Despite diverse triggering events and contexts, empirical evidence reveals four universal patterns that characterize human mobility responses across emergency types: (1) temporal evolution through distinct phases, (2) spatial reconfiguration toward localized networks, (3) adaptive emergence of new behaviors, and (4) the powerful influence of psychological factors on decision-making (Fig. 3). While we discuss these universal patterns sequentially in this section, it is crucial to recognize their deep interconnection; for instance, psychological influences often act as the underlying drivers for the observable temporal and spatial shifts, which in turn can evolve into new, lasting adaptive behaviors.

#### Temporal evolution of mobility patterns

Emergency-induced mobility follows a temporal trajectory comprising three distinct phases. The initial response phase is marked by sharp disruption of normal movement patterns^27,28^. This disruption can take one of two forms: either a mass exodus from the affected area (as seen in hurricane evacuations)^29–32^ or an abrupt standstill (as seen in pandemic lockdowns)^33–35^. For example, during the COVID-19 pandemic, public transit use in Metro Manila plummeted by 74.5%^36^, and mobility in China’s Guangdong-Hong Kong-Macao Greater Bay Area fell about 79.57% below baseline levels^37^. However, initial compliance with movement restrictions typically diminishes over time due to “lockdown fatigue”^38–41^. In Japan, for instance, public compliance decreased significantly during the third and fourth emergency declarations^39^.

Following the initial disruption, a stabilization phase emerges, characterized by gradual mobility recovery as populations adapt to changed conditions^28^. People may return to work once immediate danger passes or find safer ways to travel, causing daily movement volumes to creep back toward pre-crisis levels. The gradual return reflects an inherent resilience in human mobility^42–44^. Recovery timescales vary significantly: local events such as earthquakes and extreme weather typically require days to weeks for mobility restoration^30,45,46^, while prolonged crises, including pandemics or conflicts, necessitate months for stabilization^33,47^.

The final equilibrium phase establishes either a return to pre-crisis patterns or permanent behavioral shifts if the crisis induces lasting infrastructure or behavioral changes^48–50^. Exceptionally severe disasters can exceed the resilience capacity of a mobility system, preventing full recovery and establishing permanently altered movement patterns^51,52^.

The previous studies regarding temporal evolution show that this temporal sequence of disruption, adaptation, and recovery constitutes a fundamental “resilience triangle” pattern observed across diverse emergency contexts.

#### Spatial reconfiguration of mobility networks

Complementing the temporal dynamics, disaster conditions systematically alter spatial mobility patterns through three primary mechanisms: range contraction, network fragmentation, and centrality redistribution. Range contraction represents the most consistent spatial response, with individuals reducing travel distances and concentrating movement within familiar local areas^53^. Evidence from post-hurricane contexts^45,54,55^ and the COVID-19 pandemic^54,56^ uniformly shows a decline in average and long-distance travel, replaced by an increase in short-distance trips. This behavior is more than a simple reaction to damaged infrastructure or formal restrictions; it represents a fundamental recalibration of risk, where the perceived safety of the local environment outweighs the benefits of broader travel. The critical insight here is that disaster mobility is initially and overwhelmingly local mobility, a reality that must anchor any effective emergency response and resource distribution strategy.

The above widespread contraction at the individual level inevitably leads to network fragmentation at the collective scale. As long-range connections are exacerbated by policy restrictions during pandemics or by physical disruption, regional and national mobility systems decompose into smaller, semi-isolated sub-networks^57–59^. What is particularly revealing, however, is that these fragmented networks do not descend into chaos. Mobility flows, though truncated, often retain their characteristic heavy-tailed distributions^5,60,61^, suggesting that spatial mobility signatures are modified but retain underlying structural regularities, with individuals continuing to exhibit preferential travel to familiar or essential destinations. For governance, the persistence of these underlying regularities means disasters do not erase predictable movement patterns but rather remap them onto more localized and constrained geographies. Understanding the persistent underlying structures enables accurate modeling and prediction of flows within fragmented systems.

Finally, centrality redistribution accompanies range contraction and fragmentation, as emergencies create new dominant flows and shift the relative importance of different locations. Traditional activity centers, including central business districts and entertainment venues, experience reduced mobility, while residential areas and essential service infrastructure gain prominence through “necessity mobility” patterns^34,62,63^. During COVID-19, trips to New York’s central business district decreased by over 80% as travel demand shifted to local grocery stores, clinics, and other vital destinations^64^. Similarly, hurricane and wildfire events concentrate movement around evacuation routes and shelters^65,66^, while conflict zones restrict mobility to perceived safe zones and critical resource access points^16,67,68^.

In general, the spatial reconfiguration of human mobility during disasters is fundamentally rooted in range contraction at the individual level—a behavioral retreat to the local. When aggregated across a population, this collective action directly precipitates network fragmentation at the system level, structurally disassembling the broader mobility landscape. Concurrent with these changes is a functional redistribution of centrality, which elevates the importance of locations essential for survival. The interplay of spatial transformations effectively documents the transition from a pre-disaster “mobility of opportunity”, characterized by expansive and economically driven travel, to a crisis-driven “mobility of necessity”. Recognizing such an integrated spatial dynamic is therefore a prerequisite for designing governance strategies that can effectively support communities where and when it matters most.

#### Adaptation and emergence of new patterns

For the third type, the temporal and spatial reconfigurations detailed in the preceding sections are not random outcomes of disruption. Rather, they are the tangible results of widespread adaptive behaviors as individuals and communities develop strategies to navigate the constraints and risks of a crisis. This subsection argues that the adoption and persistence of adaptive strategies such as altered travel modes and reorganized daily activities explain how temporary shocks can evolve into a lasting “new normal”.

The mechanisms of this transformation are rooted in practical, everyday decisions. For example, the modal shifts away from public transit discussed earlier are a direct adaptive response to minimize perceived infection risk^69–71^. Similarly, the temporal adjustments that flatten commuter peaks are conscious choices made to avoid crowds^72^. Most profoundly, the spatial reorganization of life toward the local level is anchored by adaptations such as remote work and localized consumption^73–76^. These choices are not merely temporary deviations; they represent a form of crisis-induced social learning that reshapes preferences and habits.

Therefore, this “new normal” is best understood as an emergent property of these interwoven adaptations. The previously described shifts in timing and location do not occur in isolation. Instead, they are facets of a holistic response to crisis that, when solidified, forge a new, durable mobility equilibrium. Major emergencies thus act as catalysts, accelerating behavioral changes that durably alter a community’s spatial footprint and movement rhythms.

#### Psychological and emotional influences on mobility behaviors

While the preceding patterns reveal how mobility systematically evolves and reconfigures, they do not fully explain why. The underlying drivers of these decisions during an emergency are primarily psychological. The field of study is vast, but three dimensions are particularly critical for understanding mobility behaviors: risk perception, institutional trust, and place attachment. These factors fundamentally guide whether, when, and where people move, often operating independently of official guidance or objective risk assessments^77–79^.

The first is risk perception, which frequently diverges from expert assessments. Individuals often miscalculate the severity of a hazard or overestimate their personal capacity to cope, leading to delayed evacuations and preventable harm, as seen during numerous hurricanes^80,81^. Conversely, elevated risk perception can trigger preemptive behavioral responses preceding official guidance. During early COVID-19 phases, fear-driven mobility reductions occurred 2–5 days before lockdown announcements across multiple countries^82–86^. Similar anticipatory evacuation patterns emerge 48–72 h before mandatory hurricane orders^30^. While beneficial for disease containment, such spontaneous responses can create panic-driven congestion during sudden-onset disasters.

Second, beyond individual cognition, mobility decisions are mediated by institutional trust. The level of confidence communities have in government agencies and official sources is a powerful moderator of compliance with evacuation orders and mobility restrictions. Across different types of emergencies, populations with higher trust in authorities demonstrate greater adherence to guidance^87–89^. In contrast, a deficit of trust can render official instructions ineffective. In conflict zones, for example, populations may flee based on informal networks and perceived threats rather than official directives, underscoring the vital importance of credible and transparent governance during a crisis^90^.

Finally, a powerful emotional counterweight to rational risk assessment is place attachment—the deep, identity-forming bonds that connect people to a location. This attachment can compel individuals to remain in high-risk areas, even when the dangers are clear and undeniable. It is a key reason why some climate-threatened communities resist relocation despite escalating hazards^91,92^. Strong place bonds also accelerate return migration following disasters, despite ongoing risks^93^. Place attachment varies systematically across populations, with rural residents, long-term community members, and indigenous groups maintaining particularly strong territorial connections that reduce evacuation likelihood^91,94,95^. These psychological anchors explain why purely rational, risk-based evacuation models often fail to predict actual behavior, as movement decisions involve identity, belonging, and emotional geography considerations beyond physical danger assessment.

Taken together, the psychological dimensions reveal a critical insight: disaster-induced mobility is not a rational calculation of physical safety; instead, it is a complex reflection of decision-making in regard to where subjective belief, social allegiance, and emotional identity can override objective analyses. This insight has profound implications for emergency governance that necessitate a shift away from a one-size-fits-all solution. Ultimately, incorporating these human realities is fundamental to designing predictive models and mobility policies that are truly equitable and resilient.

### Manifested inequities: socioeconomic divides in disaster mobility

While universal patterns characterize mobility responses across populations, these patterns manifest very differently depending on social position. Disaster-induced mobility patterns systematically reflect underlying social hierarchies, with wealth, race, gender, age, and disability status determining evacuation capacity and survival outcomes^96–98^. The intersecting inequalities manifest as constrained, delayed, or denied movement for marginalized groups, demonstrating that disaster-affected mobility is fundamentally stratified. This section examines three primary dimensions of mobility inequality: (1) socioeconomic and racial disparities, (2) demographic vulnerabilities, and (3) digital and informational inequality.

#### Socioeconomic and racial disparities

Socioeconomic status is a primary determinant of evacuation capacity during emergencies. Low-income households frequently lack private vehicles or financial resources necessary for rapid evacuation, resulting in “forced exposure” to life-threatening conditions^99–101^. Empirical studies consistently show that economically disadvantaged and minority communities exhibit lower evacuation rates despite receiving warnings.

The intersection of poverty and racial marginalization amplifies evacuation barriers. During Hurricane Harvey, for instance, non-poor White communities evacuated at rates 19.8% above baseline, while poor Hispanic communities evacuated 12.2% below baseline despite comparable flooding^5^. In another case, low-income Latina/x/o neighborhoods disproportionately experienced climate change-attributed flooding, with 30–50% of flooded properties affected solely due to climate-enhanced precipitation^102^. While comparative analysis across multiple U.S. hurricanes reveals variation in income- and race-based evacuation disparities by event and methodology^103^, the overall evidence consistently indicates that poverty and social marginalization significantly impede effective evacuation across diverse contexts.

The above inequality patterns extend globally across different hazard types. During the COVID-19 pandemic, mobility data from multiple countries revealed that high-wealth communities had a greater capacity to reduce movement through remote work and home sheltering compared to low-wealth communities, whose members often needed to remain mobile to maintain their livelihoods^104,105^. In Chile’s 2024 wildfires in Valparaíso, analysis of evacuation records revealed that while residents from all income groups fled the fire-affected areas, those from the lowest socioeconomic strata remained displaced for the longest durations^106^. Limited socioeconomic capabilities likely impeded their return, resulting in more prolonged disruptions to their lives and livelihoods compared to wealthier evacuees who could more quickly find alternative accommodations.

Taken together, such evidence reveals a critical insight: socioeconomic advantage confers adaptive flexibility. Whether the optimal protective action is to flee a hurricane or to shelter-in-place during a pandemic, wealth affords the agency to execute that action, a privilege fundamentally denied to disadvantaged groups. These disparities create “mobility mismatches”—scenarios where a group’s capacity for movement fails to align with the spatiotemporal dynamics of a threat, exacerbating adverse outcomes for already vulnerable communities.

#### Age, gender, and disability

Meanwhile, upon closer examination of the demographic groups, we found that age, gender, and disability status create overlapping mobility constraints that compound during various disasters, with intersecting vulnerabilities often resulting in cumulative disadvantages for affected populations.

The constraints associated with age, for example, are not merely a function of chronology but are also socially mediated. For both the elderly and the young, vulnerability stems from dependency. Elderly individuals face mobility limitations arising from a confluence of chronic illness, physical impairments, and eroding social or logistical support networks^107^, a reality tragically reflected in their disproportionately high fatality rates in events such as floods^108^. Yet, this vulnerability is not static. In a seemingly paradoxical pattern, older individuals may exhibit a higher intention to migrate away from slow-onset threats such as heatwaves, demonstrating a heightened awareness of their own susceptibility^109^. In contrast, children’s mobility is almost entirely contingent on adult decision-making and capacity^110^. Crucially, these dependencies are not isolated; for families with multiple dependents, such as young children and an elderly relative, evacuation challenges are compounded, especially under resource scarcity^111,112^.

Similarly, gender shapes mobility not as a standalone attribute but through a web of social roles, economic structures, and cultural norms. While women may exhibit higher risk perception and evacuation compliance^109,113^, this is paradoxically coupled with unique constraints. The disproportionate burden of caregiving for children, the elderly, and the sick systematically delays or complicates female-led evacuations^114,115^. These social roles intersect with structural economic disadvantages, as women often have more limited access to financial resources and dedicated transportation^26^. In some socio-cultural contexts, these constraints are further solidified by norms that restrict women’s autonomy, rendering them immobile without male approval^116^. As research from Bangladesh illustrates, the threat of social sanction or harassment can be as formidable a barrier as any physical obstacle, effectively immobilizing women within their homes^117^.

Disability-related mobility barriers pose significant obstacles requiring specialized evacuation accommodations. Individuals with mobility impairments may be unable to utilize standard evacuation routes, including stairwells or crowded transportation^118^. Emergency planning frequently overlooks these requirements, leaving shelters without appropriate facilities and official communications inaccessible to many people with disabilities^119,120^.

The above evidence reveals a critical principle for mobility governance: mobility inequity during disasters is fundamentally intersectional. The demographic factors of age, gender, and disability do not operate as discrete variables with additive effects. Instead, they intersect to create a matrix of disadvantage where disadvantages are multiplicative and reinforcing. An elderly woman with a disability, for instance, confronts not three separate challenges, but a single, deeply compounded reality of marginalization where age-related frailty, gendered social roles, and an inaccessible environment converge. Consequently, a governance framework predicated on siloed, categorical thinking is inherently inadequate because it fails to address the compounded risks produced at the intersections of these identities. Achieving equitable and resilient mobility, therefore, demands a fundamental shift towards an intersectional analysis—one that interrogates the underlying social, economic, and institutional mechanisms that produce these cumulative disadvantages.

#### Digital and informational inequality

Another important aspect inevitably lies in the information access, as it systematically impedes emergency evacuation for marginalized communities. Effective evacuation depends on the timely receipt and comprehension of warnings, yet populations lacking reliable communication technology or media access face severe disadvantages. Digital divides, for instance, indicate that individuals without internet access or smartphones may miss early warnings distributed via applications or social media platforms^121^. These information gaps particularly affect vulnerable populations who often face the greatest evacuation barriers simultaneously.

Contemporary disaster response increasingly relies on big data analytics that may inadvertently reflect and reinforce representation biases. Mobility datasets derived from mobile phones, GPS systems, or social media platforms systematically over-represent certain demographics (typically younger, urban, affluent males) while under-representing others, including older adults, rural residents, low-income individuals, and women^122^. Mobile phone ownership surveys in low- and middle-income countries, for example, indicate that wealthier individuals demonstrate ownership rates three to twelve times higher than those in poorer groups^122^. This skewed representation suggests that evacuation studies based on such data may mischaracterize or omit large population segments without digital connectivity. A study in Sub-Saharan Africa showed significant gender gaps in mobile phone ownership, resulting in mobility data gaps that can undermine aid distribution and resource allocation^123^.

In summary, emergency mobility patterns reveal how structural inequities function as internal pressure points within a social system. Wealth, race, age, gender, disability, and access to information collectively determine how the impacts of an external shock propagate through different population segments. These factors compound rather than operate independently, demonstrating that mobility changes during a crisis reflect not merely individual choices but systemic vulnerabilities embedded within social structures. Without deliberate efforts to mitigate these inequities, evacuation planning may primarily benefit privileged populations, leaving the most vulnerable populations behind.

### Cascading effects across interconnected systems

**Fig. 4:**
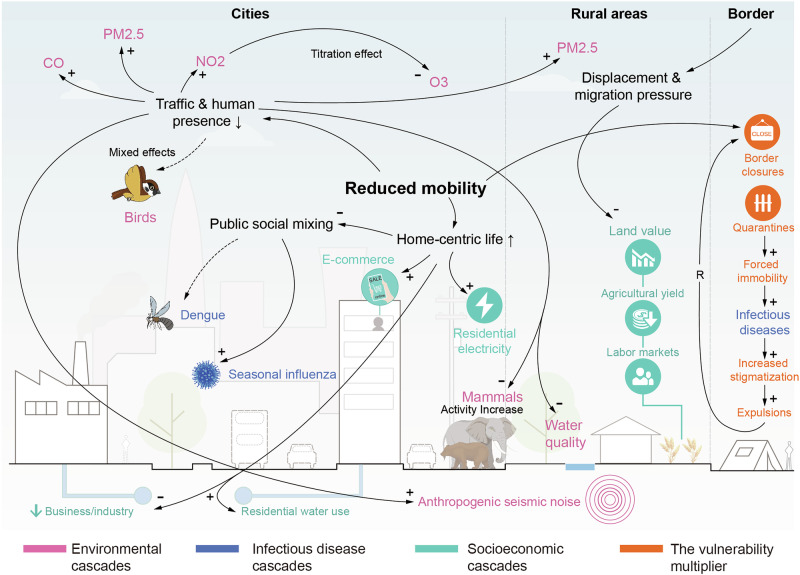
Systemic cascades triggered by emergency-induced mobility disruptions across urban, rural, and border regions. This conceptual diagram uses a system dynamics approach to map the cascading effects of altered mobility. Arrows with positive (+) and negative (−) polarity trace the causal chains from the initial shock to its multi-order impacts. In urban areas, reductions in traffic and industrial activity were associated with declines in primary pollutants (NO₂, PM₂.₅, CO), increased ozone (O₃), shifts in disease incidence (e.g., dengue, influenza), and changes in wildlife activity. Rural impacts included shifts in labor availability, agricultural yield, and land value. At borders, mobility restrictions amplified vulnerability through forced immobility, exposure to infectious diseases, and exclusionary policies. Color-coded icons represent environmental (magenta), epidemiological (blue), and socioeconomic (green) domains. The figure highlights how emergency-driven disruptions spread across sectors and spaces through tightly coupled human–environment systems. Figure created using Adobe Illustrator, version 28.7.9.

Disaster-induced mobility shifts trigger systematic transformations across interconnected environmental, health, and economic systems. These cascading effects extend far beyond immediate movement patterns, revealing how human mobility fundamentally shapes planetary and social dynamics (Fig. 4). Understanding these secondary consequences proves essential for comprehensive emergency management and demonstrates how mobility disruptions can simultaneously expose system vulnerabilities and create opportunities for sustainable transitions.

#### Atmospheric and chemical cascades

Mobility restrictions during COVID-19 created unprecedented atmospheric chemistry experiments, revealing the direct relationship between human movement and air quality. Major pollutant reductions occurred globally, with nitrogen dioxide (NO₂) levels declining 3–58% in large cities, carbon monoxide (CO) emissions falling 7.42% in China, and fine particulate matter (PM₂.₅) concentrations dropping 2.9–76.5% worldwide^124–126^, These improvements generated measurable health benefits, including over $330,000 in hospitalization cost savings in one Brazilian metropolis^127^.

However, atmospheric responses demonstrated chemical complexity beyond simple pollution reduction. Secondary pollutants such as ozone (O₃) frequently increased—rising up to 252.3% in some regions—due to reduced titration from diminished nitrogen oxide emissions^125,126,128^. Spatial disparities also emerged, with urban areas experiencing greater pollution reductions than rural areas, and socioeconomic differences affecting exposure patterns^129,130^. Migration patterns during restrictions created paradoxical outcomes, such as increased population-weighted PM₂.₅ exposure in China as urban-to-rural migration shifted populations toward regions with higher household air pollution^131^.

#### Ecological system reorganization

The global “anthropause” during COVID-19 revealed the extent to which human mobility shapes wildlife behavior and distribution^132,133^. Reduced human activity enabled rapid wildlife expansion into previously human-dominated spaces, with increased bird presence in urban areas and near transportation infrastructure^134^, and mammals venturing onto roads and into previously avoided areas^19^. These responses demonstrated the “landscape of fear” that normal human mobility creates for wildlife^135^. Wildlife responses varied significantly across species and contexts. Threatened species such as brown bears increasingly used human-dominated spaces and predicted wildlife corridors during restrictions^136^, while avian responses showed mixed patterns with both positive and negative adaptations to reduced human activity^137^. Semi-captive African elephants exhibited reduced anxiety-related behaviors when tourist visits ceased, but showed renewed stress upon visitor return^138^, suggesting chronic stress from routine human-wildlife interactions.

Marine ecosystems experienced concurrent benefits through reduced anthropogenic noise^139^, while displacement-driven fishing pressure changes demonstrated complex ecological-social interactions. Conflict-induced population movements in Uganda, for instance, increased Lake Victoria fishing effort, initially raising catches but subsequently depleting Nile perch stocks and triggering cross-border conflicts^140^.

#### Infectious disease dynamics

Mobility changes in response to disasters create natural experiments that reveal complex relationships between human movement and the transmission of pathogens. Vector-borne disease responses demonstrated particular complexity during mobility restrictions. Initial predictions suggested lockdowns would increase dengue transmission by concentrating people in residential areas^141^, yet empirical evidence showed significant dengue incidence decreases during mobility restrictions across multiple countries^142,143^. However, this initial reduction proved dynamic. Malaysia experienced initial decreases followed by an accelerated resurgence as mosquito populations adapted to new human behavioral patterns^144^, illustrating the dynamic nature of vector-host interactions.

Directly transmitted pathogen dynamics revealed mobility network dependencies through dramatic reductions in pediatric infectious diseases^145^ and seasonal influenza^146^, and parasitic conditions such as head lice when school-based transmission networks were disrupted^147^. These widespread reductions demonstrated how “normal” infection dynamics depend on specific mobility-enabled social mixing patterns.

Displacement-driven transmission acceleration contrasted sharply with restriction-induced reductions. Population displacements frequently accelerate infectious disease outbreaks through two primary mechanisms: introducing pathogens into immunologically naive populations and disrupting disease surveillance systems. Syrian refugees, for example, demonstrated markedly elevated cutaneous leishmaniasis rates compared to host communities in Jordan and Turkey^148,149^, reflecting pathogen introduction into new geographic areas. This pathogen introduction mechanism has historical precedents, with conflict-induced displacements in pre-industrial Europe closely tied to the plague spread through overcrowding and compromised immune defenses^150^. Additionally, displacement compromises disease surveillance systems, exemplified by persistently high tuberculosis incidence among displaced populations in conflict-affected Nigerian regions, where monitoring and treatment continuity are disrupted^151^.

The aforementioned observations demonstrate that mobility patterns fundamentally shape the ecological niches of pathogens. Dramatic movement changes transform entire disease landscapes through behavioral pattern reorganization rather than simple geographic shifting, revealing infectious diseases as dynamic processes intimately connected to human mobility networks rather than fixed entities.

#### Socioeconomic cascades

Emergency mobility disruptions trigger fundamental economic restructuring that extends far beyond temporary adjustments, acting as accelerants for structural transformation. Public health emergencies, for instance, have spurred a large-scale substitution of physical mobility with virtual alternatives, establishing telework, remote education, and e-commerce as predominant substitutes for travel during lockdowns. The resulting adoption of telework, remote education, and e-commerce persisted beyond acute crisis phases^152–155^, representing not temporary adjustments but a fundamental acceleration of digital transformation across sectors.

The COVID-19 pandemic revealed stark economic sector differentiation, with physical presence-dependent sectors suffering disproportionately while others demonstrated resilience through digital adaptation. Retail patterns shifted dramatically toward online channels^156^. Meanwhile, Japanese industrial analysis revealed that, despite an overall decline in GDP, the wholesale/retail, finance/insurance, and communication industries maintained stability through teleworking and e-commerce adaptations^157^. Resource consumption patterns exhibited spatial redistribution rather than proportional scaling during mobility restrictions. Water consumption shifted from commercial to residential settings without overall reduction^158^, while energy consumption patterns transformed with residential electricity usage rising up to 58.39% as transportation-related demand fell^159^. Agricultural systems demonstrated particular complexity in conflict zones, where forced displacement restructures production through cascading effects, including labor market changes, land availability shifts, and investment pattern modifications^160^.

The evidence of digital acceleration, sectoral differentiation, and resource redistribution collectively demonstrates that mobility disruptions drive economic creative destruction, simultaneously dismantling existing structures while creating space for new adaptations. Emergency-driven mobility changes function as catalysts, accelerating underlying structural transformations rather than temporary setbacks requiring recovery to previous states.

#### Vulnerability feedback loops

The final cascading effect to consider is a reflexive one, where the consequences of mobility restrictions create a feedback loop that can amplify the very vulnerabilities that initially caused mobility challenges. Emergency mobility restrictions systematically amplify pre-existing vulnerabilities, imposing disproportionate burdens on marginalized populations through cascading effects rather than affecting populations uniformly. This vulnerability multiplication occurs across multiple domains simultaneously.

Displaced population impacts proved particularly severe during COVID-19, with refugee and displaced populations experiencing border closures, quarantines, expulsions, increased stigmatization, and exclusion from health and economic relief programs^161^. The combination of forced immobility, discriminatory policies, and inadequate living conditions created compounding vulnerabilities that transformed temporary restrictions into lasting disadvantages.

Health access disruptions transformed manageable conditions into acute crises for vulnerable populations. Parkinson’s disease patients experienced increased psychological distress and worsening motor symptoms during lockdowns due to reduced healthcare service access and physical therapy disruption^162^. Such patterns reveal how mobility restrictions can compromise essential care networks, converting chronic conditions into acute health emergencies. Additionally, safety and security vulnerabilities were manifested through changes in differential crime patterns. While reported thefts decreased significantly during lockdowns, distress calls increased for crimes against women, children, and elderly individuals^163^, suggesting that mobility restrictions disproportionately endangered populations already at domestic violence risk by limiting escape options.

The body of evidence presented here establishes mobility restrictions as vulnerability multipliers that transform pre-existing disadvantages into acute crises through cascading mechanisms. This perspective challenges simplistic emergency impact assessments and emphasizes the importance of equity considerations in crisis mobility management.

## Discussion

### Overall evolutionary trend of the topic

#### From static snapshots to dynamic digital traces

From our review, it is clear that traditional mobility research relied heavily on census records and retrospective surveys, capturing only static snapshots of population distributions rather than dynamic movements. The emergence of digital data sources has fundamentally transformed our understanding by revealing continuous, high-resolution mobility patterns during crises. Mobile phone data has been particularly revolutionary, with Call Detail Records (CDRs) documenting evacuation patterns during hurricanes^68,96,164^, displacement after earthquakes^165^, and mobility contractions during COVID-19^166,167^. This transition from static snapshots to continuous observation has revealed previously invisible temporal dynamics, including anticipatory movements before official warnings^86^, phased evacuation patterns^168^, and complex return trajectories^5^.

Recent advances in research leverage multiple complementary data streams, each revealing different dimensions of crisis mobility. During COVID-19, groundbreaking studies integrated mobile phone trajectories with social media data^169,170^, transportation sensors^171,172^, socio-economic status^173,174^, and health outcomes^175,176^. This multi-source approach has exposed complex relationships between mobility transformations and their causes and consequences, moving beyond simple descriptive documentation toward a mechanistic understanding.

#### From counting numbers to analyzing networks

Early disaster-induced mobility research focused primarily on counting—evacuation rates, displacement totals, and migration volumes. While valuable, these volume metrics captured only the most superficial dimension of mobility transformations. The methodological frontier has shifted toward network and spatial metrics that reveal the structural reconfiguration of mobility patterns during crises.

Network metrics have proven particularly valuable for understanding how mobility connections transform during abnormal conditions (recall the spatial reconfiguration of mobility networks section). During COVID-19, studies employing network analysis documented how mobility restrictions reconfigured spatial interaction patterns—reducing network density^177–179^, increasing community modularity^180,181^, and transforming centrality distributions^59,182^.

Furthermore, spatial metrics have similarly advanced beyond simple displacement distances. Innovative approaches, including radius of gyration^183–185^, activity space delineation^43,186^, and spatial entropy^187,188^, capture more nuanced dimensions of mobility transformations. During COVID-19, these metrics revealed how mobility contractions were not merely quantitative reductions but qualitative transformations—with activity spaces becoming more concentrated, regular, and neighborhood-focused.

#### From reactive responses to predictive mobility management

The frontier of crisis mobility research increasingly focuses on predictive modeling—anticipating movement patterns before they occur. For pandemics, advanced models now predict likely mobility responses to policy interventions^189,190^, enabling more effective intervention design and resource allocation before implementation. For natural disasters, predictive evacuation models integrate hazard characteristics, transportation networks, and population demographics to anticipate likely movement patterns before disasters strike^191,192^.

Methodological advances across multiple domains provide the foundation for this predictive power. Machine learning approaches, including deep neural networks^193–195^, graph-based models^196,197^, and agent-based simulations^198–201^, integrate heterogeneous data sources to produce increasingly accurate mobility forecasts under novel crisis conditions^202^. The resulting models and forecasts not only enhance predictive accuracy but also enable a more robust assessment of disaster risk and resilience in coupled human-infrastructure systems^203^. Furthermore, the application of such technologies is expanding beyond pure prediction to the investigation of human-AI interaction patterns—a line of inquiry critical for shaping more effective and equitable decision-making in disaster scenarios^204^.

The predictive turn has profound implications for emergency management. Anticipatory rather than reactive response becomes possible, with resource pre-positioning, traffic management, and shelter establishment guided by expected rather than observed movement patterns. This transition from reactive to anticipatory mobility management represents perhaps the most significant practical contribution of recent methodological advances.

### Context dependency: cross-regional analyses of hazard-induced mobility

While our review identifies universal patterns that characterize mobility responses to disasters, the expression, scale, and consequences of these patterns vary significantly across different socioeconomic contexts. This section elaborates on a core finding of our review through cross-regional comparison: mobility outcomes are shaped not only by the nature of the hazard itself but are more profoundly determined by a region’s pre-existing developmental status, governance structures, and socioeconomic conditions. We term this principle “context dependency”, where the same environmental stressor catalyzes vastly different mobility outcomes in different settings, thereby explaining how universal patterns manifest at the local scale.

This principle of context dependency is particularly evident when comparing responses to major hydrometeorological events, such as hurricanes and cyclones. In a high-income country, such as the United States, the emergency system is presumed to be resilient^205^. Consequently, the critical issue exposed by a disaster is not a failure of the system itself, but the inequities in how its protective capacity is distributed. Studies consistently show that during major events such as Hurricanes Katrina, Harvey, and Irma, the ability to evacuate, as well as evacuation distance and duration, were heavily stratified by household income, race, and vehicle ownership^5,96,206,207^. For instance, wealthier, predominantly white communities not only exhibit significantly higher evacuation rates but also tend to select larger, more distant cities for shelter^30,191^. The fundamental challenge highlighted here is one of stratified resilience: the system functions, but its benefits are unequally allocated among the population.

In contrast, in a low-income country, such as Mozambique, a cyclone of comparable magnitude (e.g., Cyclone Idai) can trigger a complete systemic failure. The issue here transcends unequal access to resources and becomes a fundamental matter of systemic collapse and survival, a reality directly exacerbated by global climate injustice. Cyclone Idai resulted in over 400,000 internally displaced persons in Mozambique and the destruction of more than 223,000 homes^208,209^. The significance of the global context is illustrated by attribution studies, which quantified that the increased intensity of the storm surge due to anthropogenic climate change was directly responsible for an additional 15,000 displacements^208^. This specific evidence demonstrates that the system was overwhelmed not just by a natural hazard, but by a hazard measurably intensified by a global phenomenon. Therefore, the comparison reveals two fundamentally different narratives of disaster mobility. In the high-income context, the story is about internal social stratification within a resilient system. In the low-income context, it is about systemic collapse exacerbated by the impacts of global climate change.

A similar pattern of context dependency emerges in responses to slow-onset hazards such as drought. The mobility patterns catalyzed by drought are highly contingent on the prevailing political and economic stability of the affected region. In a relatively stable developmental context, such as Ethiopia, drought primarily functions as an economic trigger. It intensifies short-term, local, and seasonal migration as households use mobility as a direct coping strategy to secure income and manage food shortages^210^. However, in contexts marked by political instability or ethnic tensions, such as Nigeria and Syria, drought acts as a conflict accelerator. In Nigeria, climate-induced desertification has exacerbated resource scarcity, fueling violent herder-farmer conflicts and driving large-scale, conflict-based migration^211^. Similarly, the severe drought preceding the Syrian civil war is widely considered a key factor that aggravated internal tensions over resources, ultimately leading to mass displacement driven by the subsequent conflict rather than directly by the environmental hazard^212,213^.

Synthesizing these cross-regional comparisons reveals a critical conclusion: a similar environmental stressor can catalyze vastly different social tipping points, ranging from localized economic adaptation to large-scale violent conflict. Therefore, any robust model of human mobility in crisis cannot be solely hazard-centric; it should be deeply rooted in an understanding of the local socio-political and economic landscape. This finding has significant implications for global policy, suggesting that disaster response and climate adaptation strategies cannot be one-size-fits-all but should be tailored to the unique vulnerabilities and capacities of each affected region.

### The FAIR-HEART frameworks: governing the dual role of mobility in disasters

Our review establishes that human mobility during crises functions as a dual-role system: it is both a response to external shocks and a transmitter of systemic, cascading impacts. Existing disaster management frameworks, however, were primarily designed for static infrastructure and single-sector threats. Consequently, they are ill-equipped to govern the complex challenges arising from mobility’s dual role. The cascading effects synthesized in this review demonstrate that mobility disruptions propagate far beyond immediate displacement to fundamentally reshape environmental, health, and economic systems. Yet, current governance approaches largely treat mobility as a passive outcome of a crisis rather than an active driver of systemic change, creating a critical governance gap.

**Fig. 5:**
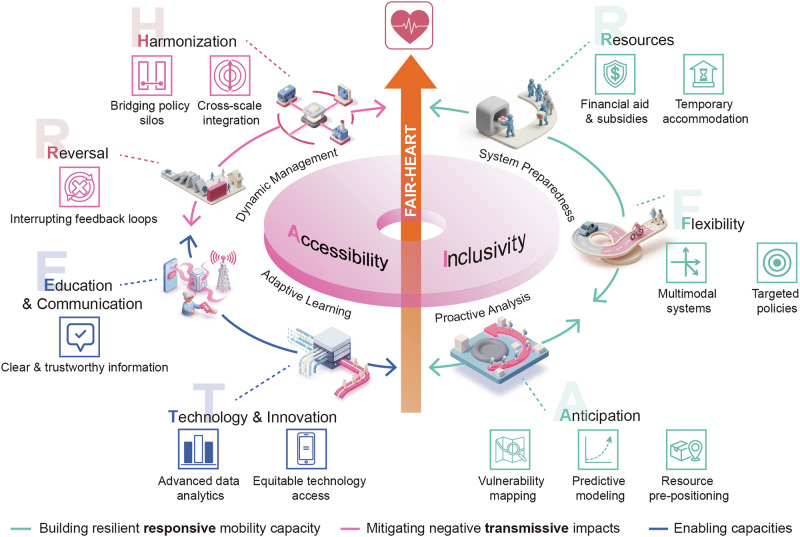
The FAIR-HEART framework. Figure created using Adobe Illustrator, version 28.7.9.

To address this gap, we propose the FAIR-HEART framework (Fig. 5), an operational model for governing the dual role of mobility that responds to recent calls for more integrated approaches to disaster risk^214^. Grounded in the people-centered, rights-based principles of the Sendai Framework for Disaster Risk Reduction 2015–2030^215^, FAIR-HEART organizes nine core principles—**F**lexibility, **A**ccessibility, **I**nclusivity, **R**esources, **H**armonization, **E**ducation, **A**nticipation, **R**eversal, and **T**echnology—into a dynamic, cyclical governance process. The framework’s ultimate objective is to leverage these principles to achieve equitable and resilient mobility outcomes.

The FAIR-HEART framework operates through an iterative, four-stage cycle built upon the foundational pillars of Inclusivity and Accessibility. These two principles function as the normative criteria against which all governance actions should be evaluated to ensure just outcomes. The cycle is structured to address the dual role of mobility: the first two stages, Proactive analysis and System preparedness, build resilient responsive capacity by leveraging Anticipation, Flexibility, and Resources; the third stage, Dynamic management, mitigates negative transmissive impacts through Harmonization and Reversal. The process concludes with the fourth stage, Adaptive learning, enabled by Education & Communication and Technology & Innovation. This final stage functions as a critical feedback loop where insights from one crisis are systematically used to refine the proactive analysis for the next, driving the continuous improvement of the governance system.

#### Inclusivity

In disaster mobility governance, the principle of Inclusivity requires moving beyond designing for the “average” person to center the lived experiences, local knowledge, and decision-making power of marginalized communities. From climate displacement to pandemic lockdowns, marginalized communities consistently report that official mobility directives fail to reflect their lived experiences^115,216^. For example, women often face greater mobility constraints due to caregiving roles, limited access to resources, and social norms restricting movement. Inadequate shelter and heightened risks of gender-based violence further hinder safe evacuation. Regional frameworks such as the Economic Community of West African States (ECOWAS) and Southern African Development Community (SADC) Gender Action Plans recognize these disparities, calling for gender-responsive evacuation and recovery systems. In contrast, community-based disaster responses that prioritize local knowledge yield to more effective strategies^115,191,217,218^. Research on environmental displacement in Bangladesh, for instance, found that community involvement resulted in more appropriate mobility interventions compared to top-down approaches^219,220^. Engaging communities as experts in their own mobility needs is therefore a strategic prerequisite for effective governance.

#### Accessibility

Accessibility demands the proactive dismantling of the physical, economic, and informational barriers that prevent vulnerable populations from utilizing mobility systems in a crisis. Traditional systems contain inherent barriers that disproportionately impact these groups. Physical barriers impede individuals with disabilities, who may be unable to use standard evacuation routes such as stairwells or find appropriately equipped shelters^221–224^. Economic barriers are a primary determinant of evacuation capacity, as low-income households often lack the private vehicles or financial means for safe passage, resulting in forced exposure to hazards^99–101^. Finally, informational barriers created by the “digital divide” can prevent individuals without internet access or smartphones from receiving critical early warnings distributed via digital platforms, a gap that disproportionately affects the elderly and low-income groups. Evidence suggests that communities with well-distributed, affordable transportation options and clear communication about available resources experience more equitable mobility outcomes^225,226^.

#### Anticipation

The principle of Anticipation requires shifting emergency management from reactive response to proactive prevention. This involves using predictive modeling, vulnerability mapping, and early warning systems to foresee mobility patterns and pre-position resources before a hazard strikes^214,227,228^. A proactive stance is essential because populations often move based on their own risk perception well before official orders are issued. For example, during the early phases of the COVID-19 pandemic, fear-driven mobility reductions occurred 2–5 days before official lockdown announcements in multiple countries^82–86^. Similarly, anticipatory evacuation patterns regularly emerge 48–72 h before mandatory hurricane orders are issued. Predictive modeling can identify these likely movement patterns, allowing authorities to manage spontaneous responses and pre-position resources, thereby improving both equity and resilience^194,229^. For example, a community-based Disaster Response Mobilization System in Puerto Rico successfully mapped household assets and vulnerabilities, which enabled grassroots organizations to mobilize resources and expedite assistance to residents with mobility issues after Hurricane Maria^230^. Such grassroots efforts affirm a foundational tenet of disaster resilience: the capacity for equitable mobility is built upon robust local preparedness^230,231^.

#### Flexibility

Flexibility is the capacity of both physical infrastructure and governing policies to adapt to the rapidly changing conditions and diverse user needs of a crisis. When primary systems are compromised, flexible infrastructure is critical. During the COVID-19 pandemic, for instance, cities with multimodal transportation options adapted more effectively than those reliant on single modes of transit. In cities such as Wuhan and New York, bike-sharing networks maintained functionality even as traditional public transit faltered, providing a crucial mobility alternative^232–235^. As crisis impacts are rarely homogenous, uniform, one-size-fits-all mobility restrictions often prove ineffective^236–238^. Research from the pandemic confirms that the effectiveness of interventions varies significantly with local economic and demographic conditions, underscoring the necessity of geographically targeted and adaptive policies^238^.

#### Resources

Where Accessibility removes systemic barriers, the Resources principle actively equips individuals with the material means to navigate a crisis, ensuring safety is not predetermined by economic status. During the COVID-19 pandemic, for example, high-wealth communities could better afford to reduce movement through remote work, while low-wealth communities, whose members often remained mobile for their livelihoods, were exposed to greater risk^104,105^. Effective governance therefore involves implementing targeted support, as financial aid, evacuation assistance, and temporary accommodation can significantly improve mobility outcomes for disadvantaged groups^115,239,240^. A clear application of this principle occurred in New York City, where a ‘Critical Workforce Membership Program’ provided essential healthcare and transit workers with free 30-day memberships to the Citi Bike bike-sharing system, offering a subsidized and safer mobility alternative^69^.

#### Harmonization

Harmonization requires integrated governance to manage the negative cross-system cascading effects of mobility shifts, directly addressing mobility’s role as a transmitter of adverse impacts. This principle mandates both horizontal coordination among sectors (e.g., transport, health, economy) and vertical integration across government scales (e.g., local, national) to prevent policy fragmentation. Our synthesis reveals why this is essential: mobility shifts trigger cascading effects across environmental, health, and economic systems. During COVID-19, for instance, uncoordinated mobility restrictions simultaneously reduced air pollution while increasing ozone levels in some regions^124–126,128^, demonstrating how siloed governance creates unintended consequences. Similarly, inconsistent policies between cities and countries created confusion and inefficiency, preventing effective management of the pandemic’s cascading effects^241^. Such a challenge is not unique to pandemics; studies on climate-induced migration consistently highlight how policy disconnection across governance scales compounds vulnerability^61,199,242,243^. Therefore, bridging these policy silos through actions such as embedding human mobility into climate and development agendas, as advocated by the Sendai Framework, is vital to prevent governance fragmentation from amplifying cascading vulnerabilities.

#### Reversal

Where Harmonization addresses broad, cross-system impacts, the Reversal principle focuses on interrupting the negative feedback loops that amplify vulnerability within affected populations over time. This principle aims to prevent temporary shocks from transforming into permanent disadvantage by actively detecting and intervening in escalating cascades of harm. Our synthesis reveals that without such intervention, mobility restrictions often create these very feedback loops. During COVID-19, for example, displaced populations experienced cascading exclusions from relief programs, and lockdowns increased domestic violence by limiting escape options—clear instances of initial shocks becoming self-reinforcing cycles of harm^161,163^. The failure to apply this Reversal principle is illustrated by the aftermath of Hurricane Maria. There, the initial mass displacement was compounded by institutional failures rooted in flawed planning and political neglect. Critical delays in restoring power and aid did not merely slow rebuilding; they created a feedback loop that perpetuated immobility and prevented the return of displaced populations, thereby transforming a temporary shock into lasting disadvantage^244^.

#### Education and communication

This principle requires the systematic creation and dissemination of clear, accessible, and trustworthy information to overcome the critical knowledge gaps that undermine crisis mobility. Information asymmetries are critical in shaping mobility outcomes during emergencies. Even well-designed systems often fail when individuals lack the knowledge to use them effectively—a gap that consistently disadvantages linguistically isolated communities, those with low digital literacy, and groups with historical distrust of authorities^245,246^. The challenge of effective information delivery is apparent in national policy. Notably, governments are increasingly incorporating mobility into DRR preparedness documents, with national strategies referencing evacuation nearly tripling between 2018 and 2023^247^. However, these documents often stop at merely acknowledging these options, while falling short of providing the detailed guidance necessary to educate the public. This highlights a critical gap between policy creation and successful communication that future preparedness efforts should address.

#### Technology and innovation

The principle of Technology and Innovation calls for a strategic governance of technology to ensure it actively promotes, rather than undermines, equitable mobility outcomes. High-tech innovations offer powerful tools for managing crisis mobility; big data analytics, mobile tracking, and digital communication platforms provide unprecedented opportunities to understand and respond to population movements^168,200,248^. However, technological solutions can also exacerbate existing inequities, particularly when digital divides are not addressed. Therefore, it is essential to combine innovative digital tools with traditional, low-tech solutions that can reach all community members, ensuring that technology serves to reduce rather than perpetuate inequalities^249^.

### Implications and limitations

This review conceptualizes human mobility during disasters as a dynamic system through which the impacts of external shocks ripple through society. Our analysis demonstrates that mobility responses under disasters exhibit universal temporal and spatial regularities. However, these responses are channeled through pathways shaped by pre-existing structural inequities. Socioeconomic status, race, gender, age, and disability function as critical pressure points that mediate the effects of a crisis, transforming individual vulnerabilities into cascading societal impacts.

Our synthesis reveals the dual role of mobility as both a consequence of disaster and an active driver of systemic change. The global mobility restrictions during the COVID-19 pandemic served as a worldwide natural experiment. They offered clear evidence of how changes in human movement can lead to profound secondary effects, such as reducing atmospheric pollution, altering wildlife behavior, and transforming disease transmission dynamics, while simultaneously amplifying pre-existing social and economic inequalities. These cascading effects demonstrate that mobility disruptions propagate far beyond immediate displacement, fundamentally reshaping interconnected environmental, health, and economic systems. In response, we developed the FAIR-HEART framework to translate these insights into governance principles that prioritize anticipatory and equitable actions over purely reactive measures.

However, critical gaps persist. The increasing reliance on digital data sources introduces significant representation biases, which can lead to skewed or incomplete understandings of mobility during crises. These datasets often underrepresent vulnerable groups, including the elderly, low-income individuals, and rural residents, thereby potentially reinforcing existing inequalities in disaster response. Furthermore, knowledge remains fragmented across disciplinary silos. Addressing these challenges is paramount. Future research should prioritize the application and refinement of integrative approaches, such as the FAIR-HEART framework, to bridge these divides. A key goal is to foster the development of more inclusive observation systems and methods that capture the full spectrum of human mobility responses, ensuring that the most vulnerable are not rendered invisible.

Finally, we acknowledge several limitations to this study. First, our review did not include gray literature, an exclusion that may result in the omission of some reports or non-academic analyses. However, previous systematic reviews suggest such omissions are unlikely to affect our principal conclusions. Second, our literature search was restricted to articles published between 2019 and our search date of July 7, 2025. This focus on the contemporary landscape, while intentional, means that foundational insights from earlier scholarship may be underrepresented. Third, our analysis did not comprehensively cover all disaster scenarios, such as slow-onset environmental changes or industrial and technological accidents. Consequently, we advise that while the principles identified here are broadly applicable, they should be applied with careful consideration to disaster contexts not extensively examined in this review. Ultimately, by understanding and governing mobility not as a mere consequence of crisis but as a central driver of societal resilience, we can better prepare for the interconnected challenges of an uncertain future.

## Methods

### Publication search procedure

We systematically searched for relevant scientific publications in the Web of Science Core Collection using the PRISMA method^250^. A query composed of two sets of keywords related to (1) human mobility and movement patterns, and (2) abnormal or crisis conditions was applied to the title and abstract fields. Details of the search strategy are provided in Table S2 of the Supplementary Information. The search was performed on July 7, 2025, covering English-language articles. This search yielded 5979 publications.

### Inclusion and exclusion criteria

The screening process involved three sequential filters applied to publications identified through database searching (*n* = 5979). First, automatically identified duplicate records (*n* = 6) were removed, yielding 5973 unique publications for screening. Second, a title and abstract screening was performed to assess topical relevance. During this stage, publications were assessed against a set of predefined inclusion and exclusion criteria. To be included, a study’s primary focus had to be on human mobility or movement patterns within the context of a disaster, crisis, or other abnormal condition, as defined by our search terms (Supplementary Table S2). Furthermore, eligible articles were limited to empirical studies, substantive theoretical contributions, or review papers published in English. Conversely, studies were excluded if they analyzed mobility under routine, non-crisis conditions or focused exclusively on non-human (e.g., animal) mobility. We also excluded studies where human mobility was treated as a minor or peripheral variable rather than a central theme, as well as publication types that do not typically present original research or synthesis, such as editorials, book reviews, and conference abstracts. Applying these criteria resulted in the exclusion of 3927 articles for lacking clear relevance. The remaining 2046 publications were provisionally classified into three categories: (1) mobility patterns under disasters (*n* = 1670), (2) cascading effects triggered by changes in human mobility (*n* = 278), and (3) review papers (*n* = 98). Third, a full-text assessment was conducted on these 2046 articles to confirm their eligibility. Articles were excluded at this stage if, upon detailed review, they did not fully meet the inclusion criteria (e.g., human mobility was a peripheral, not central, theme). This led to the exclusion of an additional 1118 publications.

**Fig. 6:**
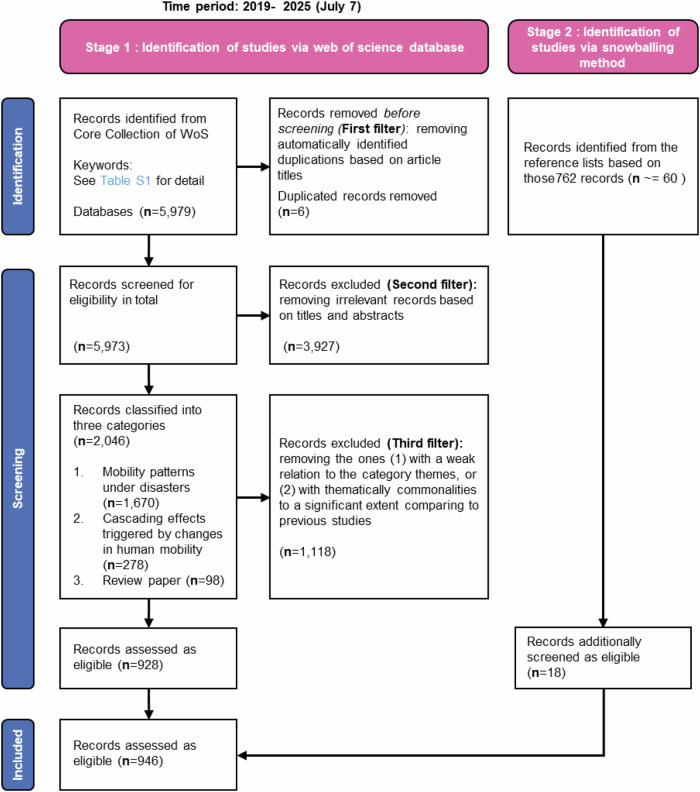
Literature screening and selection process. Figure created using PowerPoint, version 2021.

Following this three-stage screening process, 928 publications from the database search were retained. An additional 18 publications identified through snowballing met all inclusion criteria, resulting in a final sample of 946 publications for systematic review. Figure 6 shows the flowchart of the systematic literature review process.

### Data extraction and coding procedure

For the 946 included studies, we developed a structured protocol to systematically extract key information according to the categories defined in Supplementary Table S1. To ensure coding reliability and reproducibility, we implemented a rigorous procedure. First, two authors pilot-coded a random sample of 30 articles to refine category definitions and verify their consistent application. Following this refinement, at least two team members independently coded the full set of articles across six facets: data type, hazard type, spatial granularity, mobility metrics, modeling approaches, and geographic region. Any disagreements between coders were resolved through consensus-based discussion. In cases where consensus could not be achieved, a third senior author made the final determination. This robust process ensures the quality of the data presented in Table 1.

## Supplementary information

Supplementary information

## Data Availability

The minimal dataset required to replicate the findings of this systematic review is publicly available. This dataset comprises the complete reference list of the 946 studies included in the analysis and can be accessed through Figshare at: 10.6084/m9.figshare.30293995.

## Code availability

This study is a systematic review, and its findings are based on the synthesis of existing literature rather than custom code.
