# Supplementary material for: Human mobility under disasters: a systematic review and framework for equitable and resilient mobility governance
Source: NPJ Nat Hazards. 2025 Nov 7;2(1):99. doi: 10.1038/s44304-025-00153-9 (PMC12592217; doi:10.1038/s44304-025-00153-9)
Supplement: Supplementary file 1 — Supplementary information [file 44304_2025_153_MOESM1_ESM.docx]

**Supplementary Information**

Human mobility under disasters: A systematic review and framework for equitable and resilient mobility governance

In the format provided by the

authors and unedited

Table S1 Category definitions

Table S2 Key words search strategies

**Table S1 Category definitions**

| **Category** | **Definition** |
| --- | --- |
| **Type of data** |  |
| Social media data | Includes data from Twitter (geotagged tweets), Facebook, Instagram, Flickr, Volunteered Geographic Information (VGI), OpenStreetMap, and other user-generated online content. |
| Mobile phone data | Includes location tracking data from mobile carriers, anonymized Safegraph data, Google’s Community Mobility Reports/Google Mobility Data, Baidu location-based service data ,and mobility datasets derived from phone-based GPS signals. |
| Census/migration | Includes government census data, migration statistics (e.g., UNHCR refugee data), socioeconomic and demographic datasets, population displacement data, crowd density estimates, population estimates, and survey-based migration studies. |
| Flight/traffic data | Includes traffic flow datasets, transportation networks, Metro smart card data, flight logs, bike-share data, License Plate Recognition (LPR) data, and evacuation-related transportation statistics, traffic accident data. |
| Simulation | movement simulations, simulation data on crowd evacuation patterns |
| Other | Include literature reviews, interviews, and other miscellaneous sources not fitting into the above categories. |
| **Type of disasters** |  |
| Natural hazards | All rapid- or slow-onset phenomena driven by Earth processes and climate changes (storms, floods, droughts, earthquakes, wild fire, sea-level rise, hurricanes, heatwaves, extreme weather, and cyclone) |
| Technological/Man-made hazards | Accidents or failures of human-made systems (industrial fires, chemical spills, transport crashes, gas leak, crowding and trampling) |
| Health emergencies | Outbreaks and pandemics of infectious disease |
| Socio-political crises | Conflict, forced migration, civil unrest and politically driven displacement |
| **Granularity** |  |
| Individual-level movements | Data that tracks individuals’ trajectories (e.g., geotagged tweets, call-detail records) |
| Aggregate with population details | Aggregated flows of many people including demographic or census-type breakdowns |
| Aggregate without population details | Aggregated flows of many people without any population-level breakdown (e.g., flight/traffic counts) |
| mixed | Studies that combine both individual-level and aggregate data sources |
| Unknown granularity | Data source or description does not make the level of aggregation clear |
| **Mobility metric** |  |
| Volume | Counts, frequencies or rates of movements (absolute or per-capita) |
| Time | Temporal measures: durations, travel times, delays, speeds |
| Spatial | Geography/extent: distances traveled, radii of gyration, location‐based measures |
| Network | Origin–destination or graph‐based metrics: connectivity, centrality, inflow/outflow measures |
| Contextual | Covariates & side‐measures: demographics, socio-economic or environmental indicators |
| **Model** |  |
| Statistical & Econometric | Traditional statistical inference and descriptive techniques for fitting and testing relationships. |
| Spatial & Network-analytic | Models of spatial interactions, origin–destination flows, network centrality/connectivity, GIS-based. |
| Simulation & Agent-based | Computational simulations of individual or aggregate movement behaviors. |
| Machine-learning & Clustering | Supervised or unsupervised ML methods for pattern detection or prediction. |
| Qualitative & Theoretical | Non-quantitative or conceptual frameworks, case studies, literature reviews, policy/legal analyses. |
| **Regions** |  |
| Africa | The disaster occurred in Africa. |
| Americas | The disaster occurred in Americas. |
| Asia | The disaster occurred in Asia. |
| Europe | The disaster occurred in Europe. |
| Oceania | The disaster occurred in Oceania. |

**Table S2 Key words search strategies**

The following search terms (Web of Science) were used:

| **Database** | **Search strings** |
| --- | --- |
| Web of Science | (TI=("human mobility") OR AB=("human mobility") OR  TI=("human movement") OR AB=("human movement") OR  TI=("population mobility") OR AB=("population mobility") OR  TI=("mobility pattern*") OR AB=("mobility pattern*") OR  TI=("movement pattern*") OR AB=("movement pattern*") OR  TI=("population movement*") OR AB=("population movement*") OR  TI=("population displacement*") OR AB=("population displacement*") OR  TI=("mass migration") OR AB=("mass migration") OR  TI=("commuting pattern*") OR AB=("commuting pattern*") OR  TI=("crowd behavior") OR AB=("crowd behavior") OR  TI=("travel pattern*") OR AB=("travel pattern*") OR  TI=("mobility behavior") OR AB=("mobility behavior") OR  TI=("human migration") OR AB=("human migration") OR  TI=("population dynamics") OR AB=("population dynamics"))  AND  (TI=("abnormal condition*") OR AB=("abnormal condition*") OR  TI=("disaster*") OR AB=("disaster*") OR  TI=("natural disaster*") OR AB=("natural disaster*") OR  TI=("pandemic*") OR AB=("pandemic*") OR  TI=("epidemic*") OR AB=("epidemic*") OR  TI=("outbreak*") OR AB=("outbreak*") OR  TI=("flood*") OR AB=("flood*") OR  TI=("earthquake*") OR AB=("earthquake*") OR  TI=("hurricane*") OR AB=("hurricane*") OR  TI=("typhoon*") OR AB=("typhoon*") OR  TI=("tornado*") OR AB=("tornado*") OR  TI=("tsunami*") OR AB=("tsunami*") OR  TI=("wildfire*") OR AB=("wildfire*") OR  TI=("drought*") OR AB=("drought*") OR  TI=("heatwave*") OR AB=("heatwave*") OR  TI=("extreme weather") OR AB=("extreme weather") OR  TI=("emergenc*") OR AB=("emergenc*") OR  TI=("crisis*") OR AB=("crisis*") OR  TI=("conflict*") OR AB=("conflict*") OR  TI=("war") OR AB=("war") OR  TI=("terrorism") OR AB=("terrorism") OR  TI=("civil unrest") OR AB=("civil unrest") OR  TI=("mass gathering*") OR AB=("mass gathering*") OR  TI=("evacuat*") OR AB=("evacuat*") OR  TI=("hazard*") OR AB=("hazard*") OR  TI=("COVID-19") OR AB=("COVID-19") OR  TI=("SARS") OR AB=("SARS") OR  TI=("Ebola") OR AB=("Ebola") OR  TI=("infectious disease*") OR AB=("infectious disease*") OR  TI=("public health emergency") OR AB=("public health emergency") OR  TI=("lockdown*") OR AB=("lockdown*") OR  TI=("quarantine") OR AB=("quarantine") OR  TI=("social distancing") OR AB=("social distancing")) |
